# Genetic Engineering Production of Ethyl Carbamate Hydrolase and Its Application in Degrading Ethyl Carbamate in Chinese Liquor

**DOI:** 10.3390/foods11070937

**Published:** 2022-03-24

**Authors:** Naihui Dong, Siyu Xue, Hui Guo, Kexin Xiong, Xinping Lin, Huipeng Liang, Chaofan Ji, Zhiguo Huang, Sufang Zhang

**Affiliations:** 1 National Engineering Research Center of Seafood, School of Food Science and Technology, Dalian Polytechnic University, Dalian 116034, China; 15942416365@163.com (N.D.); morganxue@126.com (S.X.); 15641686835@sina.cn (H.G.); xiongkx1953@163.com (K.X.); yingchaer@163.com (X.L.); lhpdxx@126.com (H.L.); jichaofan@outlook.com (C.J.); 2 Liquor-Making Biotechnology and Application Key Laboratory of Sichuan Province, Sichuan University of Science & Engineering, Yibin 644005, China; hzgwww@126.com

**Keywords:** ethyl carbamate, ethyl carbamate hydrolase, immobilization, Chinese liquor, gas chromatography-mass spectrometry (GC/MS)

## Abstract

Ethyl carbamate (EC), classified as a Group 2A carcinogen, is most abundant in the fermented foods, such as Cachaca, Shaoxing wine, and Chinese liquor (baijiu). Although biodegradation can reduce its concentration, a high ethanol concentration and acidic environment often limit its degradation. In the present study, a novel ethyl carbamate hydrolase (ECH) with high specificity to EC was isolated from *Acinetobacter calcoaceticus*, and its enzymatic properties and EC degradability were investigated. ECH was immobilized to resist extreme environmental conditions, and the flavor substance changes were explored by gas chromatography-mass spectrometry (GC/MS). The specific enzymatic activity of ECH was 68.31 U/mg. Notably, ECH exhibited excellent thermal stability and tolerance to sodium chloride and high ethanol concentration (remaining at 40% activity in 60% (*v*/*v*) ethanol, 1 h). The treatment of immobilized ECH for 12 h decreased the EC concentration in liquor by 71.6 μg/L. Furthermore, the immobilized ECH exerted less effect on its activity and on the flavor substances, which could be easily filtrated during industrial production.

## 1. Introduction

Ethyl carbamate (EC), also known as urethane, is a chemical substance formed during fermentation and is widely abundant in many fermented foods, such as alcoholic beverages and soy sauce [1]. Animal studies have demonstrated that EC is a multi-locus carcinogen, causing lung cancer, lymphatic cancer, liver cancer, skin cancer, and breast cancer [2]. Therefore, understanding the carcinogenic mechanism of EC has emerged as a research hotspot. The carcinogenicity of EC ingested from food is mainly induced by the formation of n-hydroxy-ethyl carbamate and vinyl-ethyl carbamate through n-hydroxylation and side-chain oxidation of cytochrome P450 in mitochondria, except for the hydroxylation of most EC and direct excretion of a small part [3,4].

EC is chemically stable and hence is difficult to eliminate once formed. The effective approaches for preventing EC accumulation in fermented foods are classified into three types: physical, chemical, and biological. However, the physical treatments induce the loss of flavor substances, while the chemical treatments contribute to environmental pollution and food safety problems [5]. In this regard, the current biological methods, including the metabolic engineering routes and enzymatic degradation, have gained increasing attention and are being applied extensively to prevent EC accumulation. As the primary precursor of EC, urea could be degraded with metabolic engineering routes. For instance, *Saccharomyces cerevisiae* (*S. cerevisiae*), the principal urea producer in many fermented foods, could be modified through metabolic engineering to reduce urea formation [6]. The action mechanism is as follows: firstly, arginine is degraded to urea by arginase (CAR1), and then urea is degraded to ammonia and CO_2_ by urea amidolyase (DUR1, 2, 3). Therefore, it is speculated that knockout of the CAR1 gene or overexpression of the DUR1, 2, and 3 genes in *S. cerevisiae* might reduce the concentration of EC and urea in wine [7,8] and rice wine [6,9,10]. However, the metabolic engineering modification would lead to the waste of arginine and ammonium ions accumulation, changing the flavor of the fermented foods. Acid urease, the most common enzyme in enzymatic degradation of EC, could degrade urea into CO_2_ and ammonia. In 1976, an acid urease was first discovered in *Lactobacillus* isolated from the gastrointestinal tract of rats [11]. Afterward, many intestinal bacteria were investigated with acidic urease activity and high catalytic specificity for urea and no catalytic activity for EC [12,13,14,15,16,17,18,19]. Zhou JL et al. discovered an acid urease with the ability to degrade urea and EC simultaneously [20]. However, no significant reduction in EC was observed in rice wine. Overall, it is difficult to reduce the generated EC concentration only by regulating the precursor content. Generally, urea and EC are differentiated by their amid bonds; for instance, urea contains two amide bonds, while EC contains an amide bond and an ester bond. A vast majority of the reported EC-degrading enzymes have been amidases so far. As for the molecular structure, esterases have a greater advantage over amidases in reducing EC concentrations, but still, little attention has been paid to the study of esterases.

Chinese liquor, also known as Baijiu in Chinese, is one of the world’s oldest and most widely drunk distilled spirits. Canada, France, the Czech Republic, and Japan have stipulated that the EC concentration in distilled spirits must be less than 150 μg/L [21,22]. Chinese liquor contains a maximum of 192 μg/kg of EC, which is slightly higher than the regulation [23] (Ethyl Carbamate and Hydrocyanic Acid in Food and Beverages—Scientific Opinion of the Panel on Contaminants). Previous studies have reported that the acidic environment and high ethanol concentration of Chinese liquor affect the EC degrading ability of enzymes [5]. Therefore, establishing a highly specific ethyl carbamate hydrolase (ECH) that could degrade EC directly and tolerate the acidic environment and high ethanol concentration in Chinese liquor is highly necessary.

In this study, the enzymatic degradation of EC was applied for the first time in Chinese liquor. Briefly, a specific ECH was obtained from *Acinetobacter calcoaceticus* (*A. calcoaceticus*) through protein sequence analysis in NCBI [24]. The study results showed that the enzyme was a highly specific esterase, which could degrade EC directly without degrading urea. Additionally, the enzymatic properties of ECH and its practical application in Chinese liquor were investigated. The effects of ECH treatment on EC concentration and flavor substances in Chinese liquor were analyzed by gas chromatography–mass spectrometry (abbreviated to GC/MS, Agilent, California, USA). Overall, the ECH showed an excellent tolerance to high ethanol concentration and the acidic environment without altering the flavor of the Chinese liquor.

## 2. Materials and Methods

### 2.1. Bacterial Strains and Chemical Reagents

*Escherichia coli* BL21 (DE3) was supplied by Invitrogen (Carlsbad, CA, USA), and the plasmid pET24b was obtained from Novangen (Madison, WI, USA). A codon-optimized DNA sequence encoding ECH was synthesized by GENEWIZ (Suzhou, China). Primers synthesis was performed in Sangon Biotech (Shanghai, China). Chinese liquor (63%, *v*/*v*) was purchased from the local markets in Dalian (Liaoning, China). EC was procured from Sigma-Aldrich Co. (St. Louis, MO, USA). The inducer isopropyl-beta-D-thiogalactopyranoside (IPTG) was obtained from Shanghai Aladdin Biochemical Technology Co., Ltd. (Shanghai, China). Luria-Bertani (LB) broth (Hope Bio-Technology Co., Qingdao, China) contained: 1% tryptone (*w*/*v*), 0.5% yeast extract (*w*/*v*), 1% NaCl (*w*/*v*), pH 7.0. Trans 2K DNA Marker was purchased from TransGen Biotech (Beijing, China). Kanamycin (Kan) sulfate was purchased from Sangon Biotech (Shanghai, China).

### 2.2. Construction of Recombinant Escherichia coli (E. coli) for Overexpression of ECH

The codon-optimized ECH gene was cloned into the *NdeI* and *XhoI* sites of plasmid pET24b to construct pET24b/AcECH. Subsequently, it was transformed into the competent cells of *E. coli* BL21 (DE3) through the heat shock method. The recombinant *E. coli* BL21 (DE3) pET-24b/AcECH was allowed to grow on the LB medium supplemented with 50 μg/mL kanamycin. The transformant was selected to verify whether the target gene was inserted into the plasmid by polymerase chain reaction (PCR, pET-p1 5′-TGCTAGTTATTGCTCAGC-3′, pET-p2 5′-GCGAAATTAATACGACTC-3′) [25].

### 2.3. Expression of ECH in E. coli BL21 (DE3)

The pre-culture of *E. coli* BL21 (DE3) harboring the pET-24b/AcECH was prepared in 10 mL of LB broth containing Kan (50 μg/mL) at 37 °C for 12 h. It was cultivated at 37 °C and 200 rpm in a shaker after being inoculated into 600 mL of the same media. When the OD600 reached 0.6, the IPTG at a final concentration of 0.1 mM was added and cultivated for an additional 20 h at 20 °C. The bacterial precipitate was obtained by centrifugation (4000 rpm, 20 min).

### 2.4. Purification of Recombinant ECH and SDS-PAGE Analysis

After being re-suspended with phosphate buffer (pH 8.0), the cultured bacteria solution was disrupted by ultrasonic wave (100 W, working for 6 s, and 10 s intervals). The supernatant of the lysate was produced by centrifugation at 4000 rpm for 20 min. Subsequently, the target protein was purified by nickel column chromatography. Afterward, the recombinant ECH was purified by eluting with an elution buffer (20 mM sodium phosphate, 500 mM NaCl, 300 mM imidazole, pH 7.4). After the ultrafiltration desalting treatment, the target protein was obtained. The purified protein samples were analyzed on a 12.5% separation gel and a 4.0% concentration of gel using sodium dodecyl sulfate-polyacrylamide gel electrophoresis (SDS-PAGE). Later, the gels were stained with a solution containing 0.1% Coomassie Blue R250, 50% methanol, and 10% glacial acetic acid, and then decolored with a solution containing 7.5% glacial acetic acid.

### 2.5. Recombinant ECH Activity Assays

An enzymatic activity unit (U) of the recombinant ECH is defined as the amount of enzyme catalyzing EC hydrolysis to form 1 μM of ammonium per minute at 70 °C and pH 7.0 under atmospheric pressure [26].

The recombinant ECH activity was determined according to the previously reported method, with slight modification [26]. Briefly, 50 μL of recombinant ECH solution (or 50 μL of ultrapure water as a control group) was incubated for 15 min at 70 °C in ultrapure water containing 350 μL of EC (340 mM). The enzyme activity of the recombinant ECH was determined by measuring the change in absorbance at 625 nm during the process.

### 2.6. The Enzymatic Properties of Recombinant ECH

The ECH was dissolved in ultrapure water. The ECH activity, including temperature, thermal stability, pH, tolerance, and the effect of metal ions and EDTA, was determined using the same method as described above.

#### 2.6.1. The Optimum Temperature of Recombinant ECH

The optimum temperature of the ECH was determined by its ability to degrade EC at different temperatures (30, 37, 40, 50, 60, 70, 80, 90, and 100 °C).

#### 2.6.2. The Thermal Stability of Recombinant ECH

ECH was stored at different temperatures (20, 30, 37, 40, 50, 60, 70, 80, 90, and 100 °C) for 1 h and then reacted at the optimum temperature to obtain the thermal stability.

#### 2.6.3. The Optimum pH of Recombinant ECH

ECH activity in different pH was determined according to the previously reported method, with some modifications [27]. Briefly, the pH value was regulated under the following conditions: 0.1 M of citric acid-0.2 M Na_2_HPO_4_ (pH 3 to 8), 0.2 M of glycine-0.2 M NaOH (pH 9 to 10), and 0.05 M of borax-0.05 M Na_2_CO_3_ (pH 11).

#### 2.6.4. Effect of NaCl Concentration and Ethanol Concentration on Recombinant ECH Activity

ECH was added to various concentrations of sodium chloride (0, 5, 10, 15, 20%, *w*/*v*) solutions and ethanol (0, 10, 20, 30, 40, 50, 60%, *v*/*v*). Later, the solution was stored at 20 °C for 1 h, and the ECH activity was measured at the optimum temperature.

#### 2.6.5. Effect of EDTA and Metal Ions on Recombinant ECH Activity

Various metal ions (MnSO_4_•H_2_O, ZnSO_4_•7H_2_O, MgSO_4_•7H_2_O, FeSO_4_•7H_2_O, FeCl_3_•6H_2_O, CaCl_2_) and EDTA were added to the recombinant ECH solution to a final concentration of 1 mM. After being kept at 20 °C for 5 min, the recombinant ECH activity was measured according to the previously reported method [26].

#### 2.6.6. Substrate Specificity of Recombinant ECH

The substrate specificity of the recombinant ECH was investigated using various compounds as the substrates. The ECH activity for different substrates was performed according to the previously reported method [27], with slight modifications. In brief, the substrates (methyl carbamate, EC, butyl carbamate, and urea) were determined at a concentration of 10 g/L.

#### 2.6.7. Determination of Kinetic Parameters of an Enzyme-Catalyzed Reaction

The reaction rate of ECH in different concentration of EC (5, 10, 15, 20, 25, 30 mM) was determined. The recombinant ECH activity was measured according to the previously reported method [26]. The least-squares linear regression of the inverse substrate concentration versus the inverse velocity (Lineweaver–Burk plots) was used to obtain the enzyme kinetic parameters, and the mean values were used to calculate the V_max_ and K_m_.

### 2.7. Immobilized Recombinant ECH Preparation

The immobilized ECH was modified according to the previously reported method [28]. Briefly, the mixture was added dropwise into the hardening liquid (0.6% CaCl_2_ and 5% boric acid, *w*/*v*) for 5 h. Then, the beads were dropped into 2% (*w*/*v*) chitosan solution for 40 min.

### 2.8. The Degradation of EC in Simulation System by Recombinant ECH

The purified ECH or immobilized ECH (1000 U/L) were added to the same simulation system (10 mL ultrapure water contained 223.95 μg/L EC, pH 7.0) at 0, 3, 6, and 9 h, and the reaction system was kept at 50 °C for 12 h. Later, the beads were filtered before the newly immobilized ECH was dropped into the simulation system. The extraction of samples was performed according to the previously reported method [29]. Briefly, a total of 2 mL sample was prepared per 3 h. The EC concentration was assayed by GC/MS [30]. The oven program was as follows: held at 50 °C for 1 min, ramped up to 180 °C at 8 °C/min, and then to 210 °C at 15 °C/min and kept at 210 °C for another 5 min. Later, the samples (2 µL) were injected for GC/MS analysis.

### 2.9. The Degradation of EC in Chinese Liquor by Immobilized ECH

The immobilized ECH (1000 U/L) was added to Chinese liquor (10 mL or 1 L commercial Chinese liquor) at 0, 3, 6, and 9 h, and the reaction mixture was kept at 50 °C for 12 h. Later, the beads were filtered before the newly immobilized ECH was dropped into the simulation system. The extraction of samples and the EC concentrations were determined according to the previously reported method [29,30].

### 2.10. Determination of Volatile Organic Compounds in Chinese Liquor

The volatile organic compounds in Chinese liquor were identified according to the previously reported method [31].

### 2.11. Statistical Analysis

All the samples were prepared and analyzed in triplicate. Data were expressed as means ± SD of at least three determinations. The results were analyzed statistically using Origin 2019b software.

## 3. Results and Discussion

### 3.1. Expression of Recombinant ECH in E. coli BL21 (DE3)

**Figure 1:**
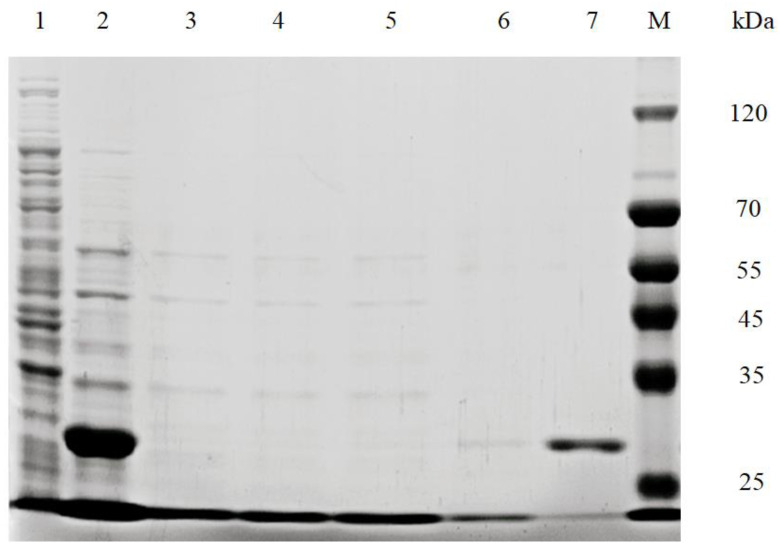
Cloning of ethyl carbamate hydrolase (ECH) and its overexpression in *E. coli*. Lane 1, *E. coli* BL21 (DE3); lane 2, whole cells of *E. coli* BL21 (DE3)/pET24b-ECH induced with IPTG; lane 3, puncture fluid; lanes 4 to 6, ECH was not eluted from a crude enzyme solution with different concentration of imidazole (20, 100, 200 mM); lane 7, ECH was eluted from crude enzyme solution with 300 mM imidazole solution (33 kDa); lane M: protein marker.

The plasmid containing the ECH gene was introduced into *E. coli* BL21 (DE3) to construct the recombinant *E. coli*. After being induced with IPTG, SDS-PAGE was performed to identify the expression of ECH. As depicted in Figure 1 lane 1 and lane 2, an empty plasmid (control group) and a crude extract was produced. A protein mass with the range of 25–35 kDa was obtained from the crude extract, indicating the successful expression of ECH.

Later, the recombinant ECH was extracted by ultra-sonication. The ECH was purified by a Ni-NTA affinity chromatography. The target protein was bound to the nickel column due to the high affinity of the six consecutive histidine residues with the immobilized nickel ions. Considering the elution, imidazole has a stronger interaction with nickel ions than the target protein [32]. As depicted in Figure 1 lane 4 to 6, the target protein was not obtained in the crude enzyme solution with different imidazole concentrations (20 mM, 100 mM, and 200 mM). Lane 7 shows the elution of target protein upon treatment with higher imidazole concentration (300 mM). The protein concentration was determined using the Bradford Protein Assay Kit (Beyotime, Shanghai, China). In this method, Coomassie Brilliant Blue G-250 combines with the alkaline and aromatic amino acids of protein, especially arginine, and changes the color of the solution to blue in the acidic medium. The color change is directly proportional to the protein concentration. Therefore, the protein concentration in the solution could be determined by detecting the absorbance at 595 nm. The purified protein concentration was 1.0 mg/mL.

The SDS-PAGE results showed that the molecular mass of the recombinant ECH was about 33 kDa (Figure 1), which was different from *Bacillus licheniformis* (42 kDa) [33], *Rhodococcus equi* (55 kDa) [34], *Bacillus paralicheniformis* (60 kDa) [26].

### 3.2. Biochemical Characterization of ECH

The Berthelot reaction [35], capable of measuring the quantity of NH_3_ generated from EC, was used to determine the ECH activity. The reaction of NH_3_ with phenol-sodium hypochlorite produces indophenol. The kinetic parameters and biochemical characteristics of the recombinant ECH were studied to investigate its potential applications. After purification using a Ni-NTA column, the specific activity of ECH containing C-terminal His-tags was found to be 68.31 ± 0.06 U/mg (70 °C).

**Figure 2:**
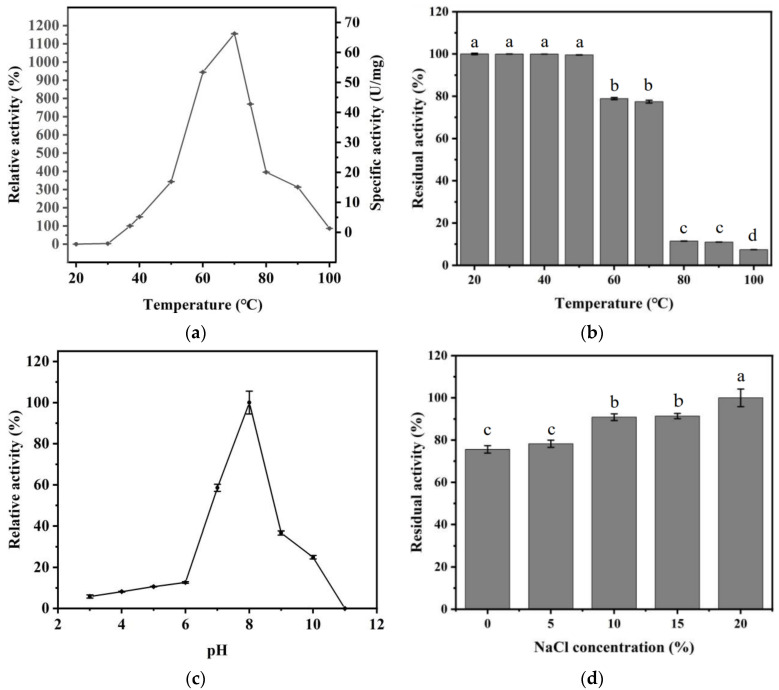

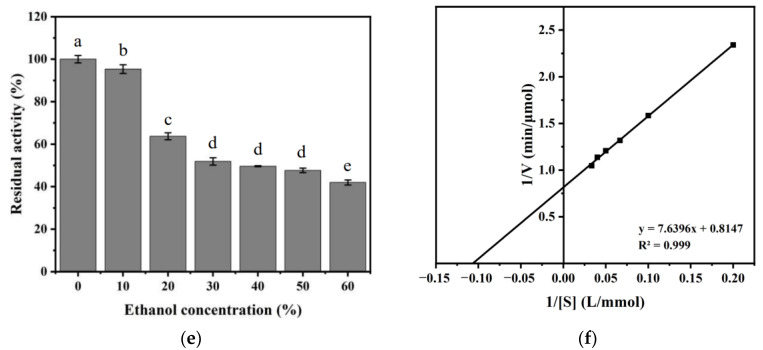
Enzymatic properties of ECH in different conditions. (**a**) Effects of temperature on ECH activity; (**b**) effects of temperature on ECH stability; (**c**) effects of pH on ECH activity; (**d**) effects of NaCl on ECH activity; (**e**) effects of ethanol on ECH activity; (**f**) Lineweaver -Burk plots of purified ECH. Letters a, b, c, d and e was used to show statistically significant differences between groups. Different letter means the significant difference, same letter means not signficant difference. (*p* < 0.05).

The ECH activity was measured at different temperatures (30, 37, 40, 50, 60, 70, 80, 90, and 100 °C), which showed an increase from 30 °C to 70 °C, peaking at 70 °C (Figure 2a). In contrast, the absorbance of the control group showed no change at 625 nm. The optimum temperature of ECH is much higher than the ethyl carbamate-degrading amidase from *A. tumefaciens* d3 (55 °C) [27] and the urethanase from *P. variabile* JN-A525 (50 °C) [36]. When ECH was located at 60 °C and 75 °C, the ECH activity reached 55.82 ± 0.05 U/mg and 45.49 ± 0.01 U/mg, respectively. As for the thermal stability, when ECH was placed at 20–50 °C for 1 h, the ECH activity remained above 99%. At 80 °C, the enzyme activity remained about 10% (7.84 U/mg). Notably, the residual enzyme activity reached 7% (5.12 ± 0.001 U/mg, Figure 2b) when it was kept at 100 °C for 1 h. The high temperature tolerance of ECH provided a unique approach for EC degradation in high-temperature fermented foods, such as Jiang-flavor liquor (the fermented temperature was 50–62 °C [37]).

Additionally, the enzyme activity of ECH under various pH conditions (3, 4, 5, 6, 7, 8, 9, 10, 11) was compared to obtain its optimum pH. The optimal reaction pH was 8.0 (Figure 2c), which was consistent with urethanase from *A. oryzae* (pH 9.6) [38] and *C. parapsilosis* (pH 10). The ECH activity reduced rapidly when the pH was less than 7.0. (Figure 2c). In many fermented foods, the acidic environment limited the application of ECH. Therefore, further molecular modification of ECH is highly necessitated to improve its properties, such as acidic environment tolerance [39].

The purified ECH was incubated with various concentrations of NaCl (0, 5, 10, 15, 20%) or ethanol buffer (0, 10, 20, 30, 40, 50, 60%) for 1 h, and then the ECH activity was measured. The residual enzyme activity increased with the increase in NaCl concentration, indicating that Na^+^ could improve the enzyme activity (Figure 2d). Meanwhile, ECH showed tolerance to high ethanol (Figure 2e) concentrations. When the ECH was placed at 20% and 40% ethanol concentration, its activity remained above 60% and 50%, respectively. Notably, after being incubated for 1 h with 60% ethanol concentration, it maintained 40% activity. Compared to previous studies, the urethanase could remain at 30% activity even after being treated with 40% ethanol for 2 h [32] and another one [27] could remain at 10% activity even after being treated with 45% ethanol for 1 h. The excellent tolerance to high ethanol concentration of ECH might reduce the EC concentration in distilled spirits.

Subsequently, the ECH activity after metal ions addition was investigated, and the results are summarized in Table 1. Most metal ions (1 mM) affected the ECH activity. Fe^3+^ and EDTA significantly inhibited the activity of carbamate hydrolase. Zn^2+^, Mg^2+^, Ca^2+^, Fe^2+^ could reduce the enzyme activity by about half. Mn^2+^ had almost no effect on enzyme activity. The substrate specificity results showed that its hydrolysis activity to methyl carbamate, ethyl carbamate, and butyl carbamate significantly decreased, which was consistent with the hydrolysis activity derived from *Lysinibacillus fusiformisstrain* (*L. fusiformis*) SC02 [33] (Table 2). The non-degrading properties of urea suggested that the ECH might be a novel carbamate hydrolase. It is known that the Michaelis constant (K_m_) represents the affinity between the enzyme and substrate. The K_m_ value of ECH for EC was 9.38 mM (Figure 2f), indicating a stronger affinity with EC than the urethanase from *L. fusiformis* SC02 (37.5 mM) and *Klebsiella pneumoniae* (74 mM) [40].

Overall, the high thermal stability and tolerance to high concentrations of ethanol and sodium chloride of ECH could provide a theoretical basis for its application in distilled spirits.

### 3.3. The Degradation of EC with Immobilized ECH in Simulation System

The severe environment of Chinese liquor (high-concentration ethanol and acidic environment) limits the EC degradation ability of most enzymes. According to Cui et al., *Lysinibacillus sphaericus* MT33, extracted from the fermented Chinese liquor grains, could decrease EC concentration by 153.04 μg/L in 3 days [29]. According to Wu et al., the immobilized *Rhodotorula mucilaginosa* were useful for rice wine (pH 3.5–4.5, 18–20% ethanol concentration, *v*/*v*), which decreased 54.18 μg/L of EC in 7 days [28]. Considering the sensitivity of ECH to the acidic environment, we immobilized the ECH (4000 U/L) to improve its EC degradation ability, which was investigated on the simulation system.

**Figure 3:**
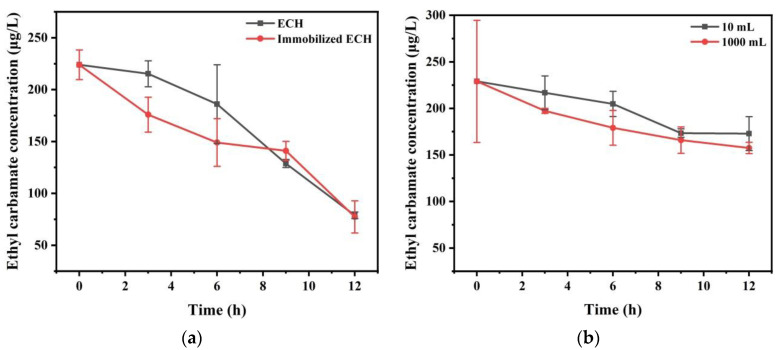
Regulatory effects of immobilized ECH on EC in simulation system and Chinese liquor. (**a**) ECH and immobilized ECH (4000 U/L) was added to the simulation system at 50 °C for 12 h; (**b**) immobilized ECH (4000 U/L) was added to different volume of the Chinese liquor (10 mL, 1000 mL) at 50 °C for 12 h.

As depicted in Figure 3a, the EC concentration (225 μg/L in initial) in the simulation system significantly decreased. The immobilized enzyme degraded 48.06 μg/L EC, which was 5.55-folds than the non-immobilized enzyme (degraded 8.66 μg/L EC in 0–3 h). However, in 6–9 h, the non-immobilized enzyme degraded 52.14 μg/L EC, while the other one degraded 2.95 μg/L. During the last 3 h, the immobilized ECH showed great ability to degrade EC similar to the first 3 h, which was about 1.2-fold higher than the non-immobilized enzyme. During 12 h treatment, the degradation efficiency of these two methods remained consistent. The immobilized ECH degraded 65.5% EC, while the non-immobilized enzyme degraded 64.8% EC.

Overall, immobilization had no effect on enzyme activity. Moreover, the immobilized enzyme could be quickly removed by filtration, which is convenient for industrial production.

### 3.4. Use ECH to Remove EC in Chinese Liquor

In the past, the degradation of EC in Baijiu was performed through inoculating fermentation. However, the alcohol content of Baijiu during fermentation is only about 10% (*v*/*v*). Therefore, controlling the EC in alcoholic beverages by enzyme could be a promising approach [37]. A high ethanol concentration in Chinese liquor reduces the EC degrading ability of enzymes. Thus, the immobilized ECH (at a final concentration of 4000 U/L) was used to eliminate the EC from Chinese liquor in a 10 mL (small) and 1000 mL (large) system and evaluate its potential industrial application.

In this study, the enzymatic degradation of EC was applied in Chinese liquor for the first time. As depicted in Figure 3b, the EC concentration significantly decreased from 229.05 ± 65.62 μg/L to 172.89 ± 18.21 μg/L in the small system (10 mL) and 157.44 ± 6.04 μg/L in the large system in 12 h. In the small system (10 mL), the EC concentration decreased by 5.38% in 0–3 h, 5.2% in 3–6 h, 13.71% in 6–9 h, and 0.23% in 9–12 h. In the large system (1 L), the EC concentration decreased by 13.85% in 0–3 h, 7.96% in 3–6 h, 5.78% in 6–9 h, and 3.68% in 9–12 h. Overall, the application of the immobilized ECH was consistent in both the systems.

Notably, the reduction trend of EC concentration was consistent irrespective of large or small systems. Compared with previous studies, *Bacillus hydrolyzae* JP21 decreased the EC concentration by 8.9 μg/kg in 7 days [41]. Similarly, another study indicated that *Lysinibacillus Sphaericus* MT33 decreased the EC concentration by 153.04 μg/L in 3 days (1 × 10^8^ CFU/kg) [29]. The different concentrations of alcohol and the processing methods had a certain influence on EC degradation in Chinese liquor. The immobilized ECH decreased the EC concentration by 71.61 μg/L (1 L Chinese liquor) or 56.16 μg/L (10 mL Chinese liquor). Herein, ECH had significant potential for degrading EC in a highly concentrated alcoholic beverage.

### 3.5. Effect of ECH on Flavor Substances in Chinese Liquor

Aside from water and ethanol, the Chinese liquor contains about 2% other compounds, such as organic acids, higher esters, alcohols, aldehydes, polyols, phenols, and other aromatic compounds [42]. Ester compounds significantly influence the primary flavor and style of liquor [38]. The flavor of Chinese liquor is dominated by esters, while other distilled liquors in the world are dominated by alcohols [39].

A total of 25 flavor substances were identified in the commercial Chinese liquor by GC/MS (Table 3), including 19 esters, 2 aromatics, 1 alcohol, 1 aldehyde, 1 acid, and 1 naphthenic. The highest content of flavor substances in both samples was ethyl hexanoate, followed by ethyl caprylate. The content of ethyl hexanoate exceeds 200 mg/L, indicating the high quality of strong-aroma liquor [43]. Compared with the non-treated sample, the contents of ethyl caproate, ethyl acetate, ethyl valerate, and furfural in the ECH-treated sample were reduced by 108.1 mg/L (from 490.58 ± 16.74 mg/L to 382.48 ± 5.27 mg/L), 6.4 mg/L (from 13.63 ± 1.10 mg/L to 7.23 ± 0.91 mg/L), 8.67 mg/L (from 22.18 ± 2.21 mg/L to 13.51 ± 1.26 mg/L), and 5.36 mg/L (from 13.92 ± 0.25 mg/L to 8.56 ± 0.25 mg/L), respectively. The remaining flavor substances remained almost constant. The threshold values of ethyl hexanoate, ethyl acetate, ethyl valerate, and furfural in air are 18.40 mg/L, 1.30 mg/L, 0.00058 mg/L, and 2.8 mg/L, respectively [44]. The total contents of these substances in the liquor were much higher than the threshold values mentioned above. Overall, the immobilized ECH treatment had no significant effect on the flavor substances detected in Chinese liquor.

## 4. Conclusions

The present study results elucidated that the established recombinant ECH had high specificity to EC. As for the enzymatic properties, the recombinant ECH exerted good tolerance to high temperature and high ethanol concentration. When ECH was stored at 100 °C or 60% ethanol (*v*/*v*) for 1 h, the specific enzyme activity of ECH reached 5.12 U/mg and 5.95 U/mg, respectively. Subsequently, the extreme environment of Chinese liquor was alleviated by embedding. The proposed method had limited effects on the enzymatic activity of ECH and the volatile organic compounds of Chinese liquor. The immobilized ECH treatment decreased the EC concentration in the liquor from 229.05 μg/L to 56.16 μg/L in 12 h.

## Figures and Tables

**Table 1:** Effect of metal cations and ethylene diamine tetraacetic acid(EDTA) on activity of ethyl carbamate hydrolase (ECH).

Metal Ion Chemical	Relative Activity (%)	Standard Deviation (SD)
Zn^2+^	56.71	1.92
Mg^2+^	64.99	2.55
Ca^2+^	58.48	1.54
Mn^2+^	96.80	2.42
Fe^2+^	58.66	5.22
Fe^3+^	10.32	1.63
EDTA	35.40	4.78

**Table 2:** Substrate specificity of ECH.

Substrate	Relative Activity (%)	SD
Methyl carbamate	132.04	0.57
Ethyl carbamate	100.00	1.30
Butyl Carbamate	80.34	0.78
Urea	0.00	1.87

**Table 3:** Volatile organic compounds untreated and treated for 12 h in Chinese liquor.

	Compounds	Concentration (mg/L)	
		Control	12 h
Esters	ethyl acetate	13.63 ± 1.10	7.23 ± 0.91
	butanoic acid, ethyl ester	29.45 ± 0.52	19.20 ± 0.33
	pentanoic acid, ethyl ester	22.18 ± 2.21	13.51 ± 1.26
	hexanoic acid, ethyl ester	490.58 ± 16.74	382.48 ± 5.27
	hexanoic acid, propyl ester	5.248 ± 0.10	4.27 ± 1.26
	heptanoic acid, ethyl ester	35.14 ± 0.87	31.61 ± 0.94
	hexanoic acid, butyl ester	20.42 ± 0.28	20.34 ± 0.50
	octanoic acid, ethyl ester	58.77 ± 1.32	58.62 ± 1.15
	isopentylhexanoate	13.31 ± 0.21	15.18 ± 0.30
	hexanoic acid, pentyl ester	9.06 ± 0.25	11.00 ± 0.14
	nonanoic acid, ehtyl ester	2.74 ± 0.04	4.37 ± 0.04
	hexanoic acid, hexyl ester	37.67 ± 0.92	50.39 ± 0.97
	decanoic acid, ethyl ester	6.69 ± 0.19	13.27 ± 0.27
	heptanoic acid, heptyl ester	1.53 ± 0.04	4.28 ± 0.16
	octanoic acid, hexyl ester	1.35 ± 0.01	4.43 ± 0.07
	tetradecanoic acid, ethyl ester	2.20 ± 0.12	12.31 ± 0.66
	hexadecanoic acid, ethyl ester	7.71 ± 2.22	33.82 ± 1.41
	9-octadecenoic acid (z)-, eicosyl ester	0.48 ± 0.05	6.74 ± 0.21
	ethyl iso-allocholate	0.27 ± 0.05	4.02 ± 0.38
Aromatic	benzeneacetic acid, ethyl ester	3.44 ± 0.09	4.38 ± 0.08
	benzenepropanoic acid, ethyl ester	2.43 ± 0.13	6.77 ± 4.86
Alcohol	1-pentanol	3.06 ± 0.18	3.85 ± 0.27
Aldehyde/furan	furfural	13.92 ± 0.25	8.56 ± 0.25
Organic acid	hexanoic acid	29.20 ± 0.82	28.51 ± 0.13
Other	butane,1,1-diethoxy-3-methyl-	0.69 ± 0.41	0.78 ± 0.07

## Data Availability

The data that support the findings of this study are available from the corresponding author upon reasonable request.

## References

[1] Jiao Z., Dong Y., Chen Q. (2014). Ethyl Carbamate in Fermented Beverages: Presence, Analytical Chemistry, Formation Mechanism, and Mitigation Proposals. Comprehensive Reviews in Food Science and Food Safety.

[2] Field K.J., Lang C.M. (1988). Hazards of Urethane (Ethyl Carbamate): A Review of the Literature. Lab. Anim..

[3] Park K.-K., Liem A., Stewart B.C., Miller J.A. (1993). Vinyl Carbamate Epoxide, a Major Strong Electrophilic, Mutagenic and Carcinogenic Metabolite of Vinyl Carbamate and Ethyl Carbamate (Urethane).

[4] Sakano K., Oikawa S., Hiraku Y., Kawanishi S. (2002). Original Contribution Metabolism of Carcinogenic Urethane to Nitric Oxide Is Involved in Oxidative Dna Damage.

[5] Zhao X., Du G., Zou H., Fu J., Zhou J., Chen J. (2013). Progress in preventing the accumulation of ethyl carbamate in alcoholic beverages. Trends Food Sci. Technol..

[6] Akinyanju J.A., Oyedeji B.M. (1993). Comparative Study of Mixed Flora and Single Strain Fermentation in the Production of Plantain (Musa Paradisiaca) “Wine”. Chem. Mikrobiol. Technol. Lebensm..

[7] Wu D., Li X., Shen C., Lu J., Chen J., Xie G. (2014). Decreased Ethyl Carbamate Generation during Chinese Rice Wine Fermentation by Disruption of CAR1 in an Industrial Yeast Strain. Int. J. Food Microbiol..

[8] Guo X.W., Li Y.Z., Guo J., Wang Q., Huang S.Y., Chen Y.F., Du L.P., Xiao D.G. (2016). Reduced Production of Ethyl Carbamate for Wine Fermentation by Deleting CAR1 in Saccharomyces Cerevisiae. J. Ind. Microbiol. Biotechnol..

[9] Wu D., Li X., Lu J., Chen J., Zhang L., Xie G. (2015). Constitutive Expression of the DUR1,2 Gene in an Industrial Yeast Strain to Minimize Ethyl Carbamate Production during Chinese Rice Wine Fermentation. FEMS Microbiol. Lett..

[10] Wu D., Xie W., Li X., Cai G., Lu J., Xie G. (2020). Metabolic Engineering of Saccharomyces Cerevisiae Using the CRISPR/Cas9 System to Minimize Ethyl Carbamate Accumulation during Chinese Rice Wine Fermentation. Appl. Microbiol. Biotechnol..

[11] Moreau M.-C., Ducluzeau R., Raibaud P. (1976). Hydrolysis of Urea in the Gastrointestinal Tract of “Monoxenic” Rats: Effect of Immunization with Strains of Ureolytic Bacteria.

[12] Suzuki K., Benno Y., Mitsuoka T., Takebe S., Kobashi K., Hase J. (1979). Urease-Producing Species of Intestinal Anaerobes and Their Activities.

[13] Yang L.Q., Wang S.H., Tian Y.P. (2010). Purification, Properties, and Application of a Novel Acid Urease from *Enterobacter* sp.. Appl. Biochem. Biotechnol..

[14] Mora D., Fortina M.G., Parini C., Ricci G., Gatti M., Giraffa G., Manachini P.L. (2002). Genetic Diversity and Technological Properties of Streptococcus Thermophilus Strains Isolated from Dairy Products. J. Appl. Microbiol..

[15] Mora D., Maguin E., Masiero M., Parini C., Ricci G., Manachini P.L., Daffonchio D. (2004). Characterization of Urease Genes Cluster of Streptococcus Thermophilus. J. Appl. Microbiol..

[16] Mora D., Monnet C., Parini C., Guglielmetti S., Mariani A., Pintus P., Molinari F., Daffonchio D., Manachini P.L. (2005). Urease Biogenesis in Streptococcus Thermophilus. Res. Microbiol..

[17] Zotta T., Ricciardi A., Rossano R., Parente E. (2008). Urease Production by Streptococcus Thermophilus. Food Microbiol..

[18] Kakimoto S., Okazaki K., Sakane T., Imai K., Sumino Y., Akiyama S.I., Nakao Y. (1989). Isolation and Taxonomic Characterization of Acid Ureaseproducing Bacteria. Agric. Biol. Chem..

[19] Chen Y.-Y.M., Clancy K.A., Burne R.A. (1996). Streptococcus Salivarius Urease: Genetic and Biochemical Characterization and Expression in a Dental Plaque Streptococcus. Infect. Immun..

[20] Zhou J., Kang Z., Liu Q., Du G., Chen J. (2016). Degradation of Urea and Ethyl Carbamate in Chinese Rice Wine by Recombinant Acid Urease. Chin. J. Biotechnol..

[21] European Food Safety Authority (EFSA) (2007). Ethyl Carbamate and Hydrocyanic Acid in Food and Beverages—Scientific Opinion of the Panel on Contaminants. EFSA J..

[22] Lachenmeier D.W., Lima M.C.P., Nóbrega I.C.C., Pereira J.A.P., Kerr-Corrêa F., Kanteres F., Rehm J. (2010). Cancer Risk Assessment of Ethyl Carbamate in Alcoholic Beverages from Brazil with Special Consideration to the Spirits Cachaça and Tiquira. BMC Cancer.

[23] Wu P., Pan X., Wang L., Shen X., Yang D. (2012). A Survey of Ethyl Carbamate in Fermented Foods and Beverages from Zhejiang, China. Food Control.

[24] Yoshida K., Takamasa T. (2020). Decomposition of Urethane.

[25] Wang J., Zhang S., Tan H., Zhao Z. (2007). PCR-Based Strategy for Construction of Multi-Site-Saturation Mutagenic Expression Library. J. Microbiol. Methods.

[26] Liu Q., Chen Y., Yuan M., Du G., Chen J., Kang Z. (2017). A Bacillus Paralicheniformis Ironcontaining Urease Reduces Urea Concentrations in Rice Wine. Appl. Environ. Microbiol..

[27] Kang T., Lin J., Yang L., Wu M. (2021). Expression, Isolation, and Identification of an Ethanol-Resistant Ethyl Carbamate-Degrading Amidase from Agrobacterium Tumefaciens D3. J. Biosci. Bioeng..

[28] Wu Q., Zhao Y., Wang D., Xu Y. (2013). Immobilized Rhodotorula Mucilaginosa: A Novel Urethanase-Producing Strain for Degrading Ethyl Carbamate. Appl. Biochem. Biotechnol..

[29] Cui K., Wu Q., Xu Y. (2018). Biodegradation of Ethyl Carbamate and Urea with Lysinibacillus Sphaericus MT33 in Chinese Liquor Fermentation. J. Agric. Food Chem..

[30] Xia Q., Yuan H., Wu C., Zheng J., Zhang S., Shen C., Yi B., Zhou R. (2014). An Improved and Validated Sample Cleanup Method for Analysis of Ethyl Carbamate in Chinese Liquor. J. Food Sci..

[31] Liang H., He Z., Wang X., Song G., Chen H., Lin X., Ji C., Zhang S. (2020). Bacterial Profiles and Volatile Flavor Compounds in Commercial Suancai with Varying Salt Concentration from Northeastern China. Food Res. Int..

[32] Crowe J., Döbeli H., Gentz R., Hochuli E., Stiiber D., Henco K. (1994). 6xffis-Ni-NTA Chromatography as a Superior Technique in Recombinant Protein Expression/Purification. Protoc. Gene Anal..

[33] Zhao C., Kobashi K. (1994). Purification and Characterization of Iron-Containing Urethanase from Bacillus licheniformis. Biol. Pharm. Bull..

[34] Akutsu-Shigeno Y., Adachi Y., Yamada C., Toyoshima K., Nomura N., Uchiyama H., Nakajima-Kambe T. (2006). Isolation of a Bacterium That Degrades Urethane Compounds and Characterization of Its Urethane Hydrolase. Appl. Microbiol. Biotechnol..

[35] Kobashi K., Takebe S., Sakai T. (1990). Urethane-hydrolyzing enzyme from *Citrobacter* sp.. Chem. Pharm. Bull..

[36] Zhou N.-D., Gu X.-L., Tian Y.-P. (2013). Isolation and Characterization of Urethanase from Penicillium variabile and Its Application to Reduce Ethyl Carbamate Contamination in Chinese Rice Wine. Appl. Biochem. Biotechnol..

[37] Jianqing D. (2017). The Process Optimization of Maotai Liquor Fortified High Temperature Daqu and Its Comparative Research.

[38] Masaki K., Fujihara K., Kakizono D., Mizukure T., Okuda M., Mukai N. (2020). Aspergillus Oryzae Acetamidase Catalyzes Degradation of Ethyl Carbamate. J. Biosci. Bioeng..

[39] Liu X., Fang F., Xia X., Du G., Chen J. (2016). Stability Enhancement of Urethanase from Lysinibacillus Fusiformis by Site-Directed Mutagenesis. Chin. J. Biotechnol..

[40] Bu P., Chen J., Du G. (2014). Purification and Characterization of a Halophilic Urethanase from Klebsiella Pneumoniae. Chin. J. Biotechnol..

[41] Xia D., QiaoYu L., Fang L., WeiZhu Z., Jian C., GuoCheng D., Fang F. (2018). Isolation of Microbial Strains for Degradation of Etliyl Carbamate in Luzhouflavour Baijiu and Characterization of Corresponding Enzymes. Food Ferment. Ind..

[42] Zhou Q., Zhang S., Li Y., Xie C., Li H., Ding X. (2011). A Chinese Liquor Classification Method Based on Liquid Evaporation with One Unmodulated Metal Oxide Gas Sensor. Sens. Actuators B Chem..

[43] Liu Q., Yao X., Liang Q., Li J., Fang F., Du G., Kang Z. (2018). Molecular Engineering of Bacillus Paralicheniformis Acid Urease to Degrade Urea and Ethyl Carbamate in Model Chinese Rice Wine. J. Agric. Food Chem..

[44] van Gemert L.J. (2011). Compilations of Odour Threshold Values in Air, Water and Other Media.

